# Histopathological Studies on Stunting Syndrome in Broilers, Lahore, Pakistan

**DOI:** 10.1155/2013/212830

**Published:** 2013-07-18

**Authors:** Muhammad Fiaz Qamar, Hina Aslam, Nusrat Jahan

**Affiliations:** Department of Zoology, GC University, Lahore 64000, Pakistan

## Abstract

Runting stunting syndrome (RSS) is a multifactorial disease with many names and faces that had caused considerable economic losses to poultry through reduced uniformity, reduced livability, decreased body weights, elevated feed conversions, and many secondary diseases. The aim of the current study was to evaluate the effect of stunting syndrome on histopathology in chicks (*n* = 120) of different ages collected from nine different farms. Grouping was done on the basis of age (G1 = 1–10 days, G2 = 11–20 days, G3 = 21–30 days, and G4 = 31–40 days) including both stunted and normal chicks. Histopathological findings were the intestinal lesions (29%), including degeneration of villi, crypts, epithelial cells and lamina propria. Pancreatic histopathological lesions (16.65%) included the fibrosis, vacuolation, and degeneration of acinar cells. Degeneration of follicles and epithelial cells, of bursa of fabricius (43%) and dilation of glandular cells of proventriculus including lymphocytes infiltration (5.6%) were other histopathological findings. All these changes may interfere with normal digestive processes and normal body functioning resulting in poor weight gain and retarded growth or stunting of chicks.

## 1. Introduction

High demand for chicken meat, early marketing age, rapid return of broiler farms, and high profit margins in the lowest possible time have increased the popularity of poultry farming. Some of the factors that are important for poultry farming are the flock size, age, and weight of chick at the time of marketing, feeding, feed conversion ratio, uniformity, and mortality. In poultry, an unusually small chick is referred as “runt” which means smallest of the flock, and retardation or hindering of normal growth comes under the term “stunting.” A condition in which members of a flock appear with relatively smaller bodies due to retarded growth is called runting stunting syndrome (RSS) [1].

Runting stunting syndrome (RSS) was first described in 1940s, then became popular to the commercial industry and has been reported variously around the world since 1970s [2]. RSS had caused economic hardships in the poultry industry through reduced uniformity, reduced livability, decreased body weights, elevated feed conversions, and many secondary diseases [3]. Many descriptive terms have been used for this disease as helicopter disease [4]; brittle bone disease [5]; pale bird syndrome [6]; and malabsorption syndrome [7]. When diseased birds are necropsied, generally inflamed stomachs (proventriculi), reduced smaller liver size but enlarged, gall bladders may appear. Thin, enlarged and translucent intestines were found, lumen of which may contain large amounts of fluids [8].

Histopathologies of affected organs or histological lesions are a clear indication for which organ is affected and to what extent? Is there any link between affected (damaged) organs, normal body functioning and/or normal body weight of an organism, clinical signs, morphological indications, and histological lesions correlate or not? In natural outbreaks, early pathological changes were observed in crypts of Lieberkuhn in most of the cases of 1st week. These changes include the cystic dilation, necrosis and degeneration of epithelial lining, accumulation of cellular debris and sometimes loss of crypts, while pancreatic atrophy has first been reported on 11th day [9]. Although the disease may be infectious in nature yet some noninfectious agents cannot be ignored. There are a large number of possible reasons for RSS including some management issues, environmental challenges, infectious diseases, faulty poultry genetics, nutrition and feeding, and perhaps some other reasons and/or any possible combinations of all these [1]. Some chicks were more susceptible than others, and the males were found much severely affected than females [3]. 

Management, nutrition, and environment play an important part in the onset of this syndrome. Infectious agents mostly viruses have been isolated as etiological agents, specifically rotaviruses, reoviruses, astroviruses, and some other small rounded viruses present in the intestines and intestinal contents of infected chicks by using some isolation techniques or direct electron microscopy [10]. Secondary bacteria are often isolated from the intestines of RSS affected birds [2]. The objective of the present study was to study the histopathological changes due to runting stunting syndrome.

## 2. Materials and Methods

### 2.1. Study Area and Duration

The present study was conducted during the period of January 2012 to July 2012 on the stunted chicks of one to forty days of age. These chicks, were procured randomly from ten different commercial poultry farms around Lahore, Pakistan. 

### 2.2. Experimental Design

Collected chicks (120) were divided into four groups; each containing 30 chicks on the basis of different ages. 18 stunted and 12 normal chicks were taken in each group. Total number of groups, age of chicks and total number of chicks in each age group are given in Table 1. 

### 2.3. Maintenance and Care

Chicks of different age groups were collected from different locations (farms) at different times where they were provided with standard poultry feed and fresh water *ad libitum* Feed of chicks were formulated by specified nutrient levels that were needed for optimum performance. Fresh water and optimum temperature (27–37°C) were also provided to chicks. Nearly similar optimum conditions were found at each farm from where the chicks were collected. 

### 2.4. Recording Clinical Signs

All the experimental chicks were recorded periodically for the clinical signs, including body weight, abnormal and malposition feathering, loss of feathers from neck and shoulders, uneven growth rate and poor performance, beaks and legs color, lameness and reluctance to move, and uneven food conversion ratios. Chicks were weighed before dissecting by using a weighing scale. 

### 2.5. Histopathological Studies

Sacrificed animals were laid on dissecting board with ventral sideupward. The fur of animal was removed with the help of a razor. From the abdominal wall of animal, an incision was made. From the abdominal region the body and thus the internal organs were exposed. From the digestive system proventriculus, intestines were separated and bursa was also removed gently. Pancreas was also separated from intestine. All the organs are rinsed in 0.85% saline solution before fixing.

### 2.6. Processing for Histological Studies

Tissues from proventriculus, gizzard, small intestine, large intestine, and kidney were removed. They were fixed in Bouin's liquid for 24 hours. Then they were dehydrated in the ethanol series, cleaned with xylol, and embedded in paraffin. Thin sections were prepared (5–7 *μ*m) cells and stained with H&E stain. All the prepared slides were studied by light microscopy, and photographs were prepared for general microscopic studies. 

### 2.7. Statistical Analysis

Mean body weights with standard errors were calculated for different groups of RSS affected chicks and normal chicks [11].

## 3. Results and Discussion

In present study, chicks of different age groups, with smaller size and retarded growth were observed. In present study, the body weights of stunted chicks were compared with normal chicks of the same age, and the mean live body weights of stunted chicks were found significantly reduced (32%) as compared to the normal chicks of same age (1–40 days old). In G1 (1–10-day-old chicks), the mean body weights observed for stunted chicks was found significantly lower (36%) as compared to normal. Similarly in G2 (11–20-day-old chicks), G3 (21–30-day-old chicks), and G4 (31–40-day-old chicks) stunted chicks were also found with 33%, 28.3%, and 31.9% reduced body weights as compared to normal members of each respective group. Decreased weight gain was also previously observed by many researchers [7, 11, 12]. Some investigators also have reported about 30 points reduced body weights in RSS cases or when the chicks were raised on built up litter from RSS affected farms [1]. Severe runting less than a third of lower body weights were also seen in affected flocks. The mean live weights of stunted chicks in affected flocks were found 31–37% of normal chicks in field observations while significant weight reductions (42–7% and 35–38%) were observed in experimental cases of chicks inoculated with whole intestinal homogenates and bacterial free intestinal homogenates [13], Table 2. These field observations were found in agreement with the results of present studies.

In 5-day- and 14-day-old chicks inoculated intestinal homogenates caused significant mean live weight reductions (23.8% and 17.7%), while inoculating with pancreatic homogenates significant mean live weight reduction (8.8%) was also seen [9]. These observations were not similar to our findings because inoculated chicks might receive various tissue homogenates of infected chicks as compared to naturally affected RSS chicks observed in this study. In present study, in addition to smaller size and recued body weights other clinical signs were also noted. Feather development was also found poor. Pale bodies with signs of listlessness and diarrhea were observed. Stunted chicks were found active and difficult to catch [7, 14, 15]. Most (but not all) of the stunted chicks exhibited paleness and poor feather development with broken shafts [14]. Elevated food conversions and poor feathering was also observed [7]. The chickens with infectious stunting syndrome were observed very active as compared to their pen-mates [15]. All these informations were found in agreement with the observations provided in present study. Most of the RSS affected chicks as studied in the present case were characterized by diarrhea and/or growth depression [16], poor feather development, lower body weight, and voracious appetites as observed in RSS chicks [17]. The syndrome had been described as the disease with many names and faces, so mostly the names are given on the basis of clinical presentations. Due to abnormal or retarded feathering in stunted chicks, the disease is known as helicopter disease, while due to pale bodies of stunted chicks the disease is sometimes referred as pale bird syndrome. Different histopathological features in stunted chicks were observed during the whole study, which include intestinal lesions, pancreatic lesions, lesions and atrophy of bursa, and some histopathological changes in proventriculus of only few cases. The unique histological features of RSS pathology in chicks were first presented by Goodwin et al., in the United States [6].

**Figure 1:**
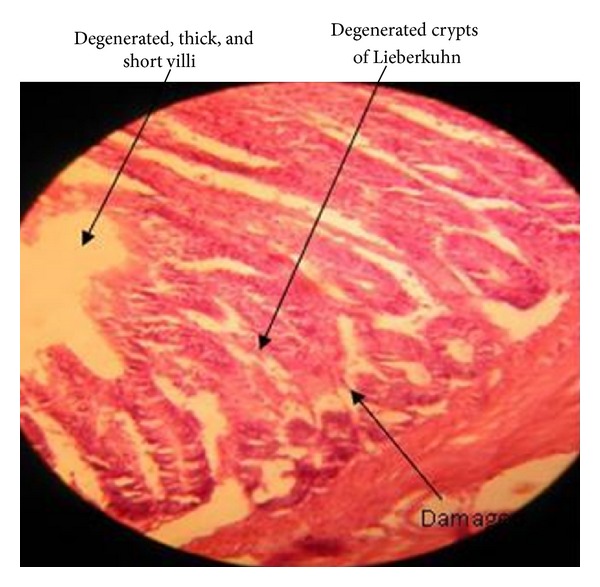
Intestine of stunted chick.

The present study reveals the intestinal lesions (29%) in 1–40-day-old stunted chicks. Various histopathological features of intestine include damaged intestinal villi; lamina propria and formation of multiple cysts on intestine including crypts. Four groups were studied to note the intestinal lesions. In all the groups stunted chicks were found to have thin and translucent intestines with more or less intestinal lesions, (Figure 1) whereas healthy chicks were found with normal intestines and no such lesions were observed in any case. Intestinal lesions were observed most frequent in 1–20-day-old chicks (44.44%) as compared to chicks of 21–30 days (16%) or 31–40 days (11.1%) of age. Occurrence of lesions was observed in chicks of four days of age and severity of lesion increased after that. But occurrence and intensity of lesions decreased as the chicks became more than twenty days of age. These intestinal changes were also observed by many other researchers. Intestinal histopathology in young chickens with stunting syndrome had been noted by several scientists [4, 9, 18, 19]. Microscopic intestinal lesions including presence of various cysts with clubbing and fusion of intestinal villi were also observed [1]. Infection was found evident during the 1st week of syndrome. Histopathological changes include the dilation of crypts of Lieberkuhn, degeneration of crypt epithelial cells, cellular debris accumulation, and/or loss of crypts [9]. Cystic changes in crypts of Lieberkuhn due to RSS were also described by kouwenhoven et al. that were found in agreement with the present study [4]. Frazier and Reece [20] also observed the most frequent changes in the lamina propria, and some cystic changes in crypts [21]. These observations were found similar to our findings on intestinal lesions. Injury and infections sometimes result in the common reactions as subsequent cyst formation and infiltration of lymphocytes into lamina propria [22]. So the resultant changes as observed in our case may be due to some underlying infection that reduces the absorptive capacity causing diarrhea. The intestinal lesions characterized by severe villous atrophy, changes in surface epithelium, and villi in RSS affected birds may account for clinical signs of diarrhea. Severity of lesions and clinical signs depend upon the extent of loss of particular intestinal glands especially enterocyte [16]. The mature enteroabsorptive cells lining the intestinal villi may be a target for infectious agents (viruses) to be destroyed. Because of degeneration or loss of enterocytes from this cell lining nutrient digestion by enzymes as well as absorption of electrolytes and nutrients is interrupted resulting in maldigestion, malabsorption, and osmotic retention of water [23]. Lesions were observed in 50% of the RSS affected flocks, but only 29% cases of intestinal lesions were recorded in current study [16]. The reason may be that the chicks were in different phases of the disease and recovery. In field cases of 3–27 days of age, intestinal histopathological changes (22.22%) in stunted chicks include distension of crypts of Lieberkuhn and villous atrophy, while incidence of cystic crypts in experimental cases was also found (57.14%). It was also found that severe intestinal lesions were present in 3–14 days of chicks, and then severity of lesions was reduced [13]. Filed observations (22.22%) were found almost similar to our findings for intestinal lesions (22.22%) in stunted chicks, Table 3.

**Figure 2:**
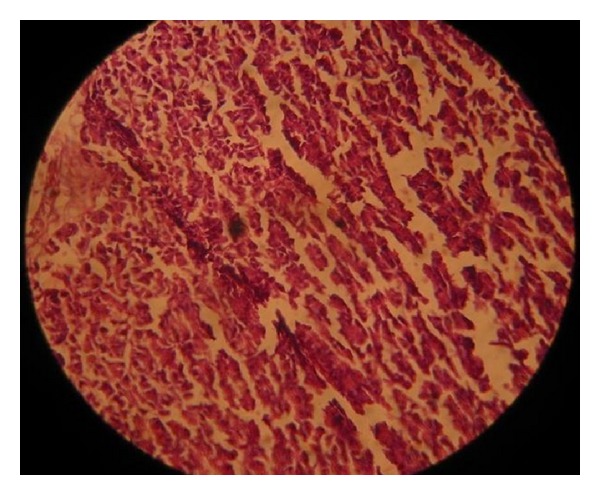
Pancreas of stunted chick showing fibrosis.

Another histopathological finding in our study was the degeneration and vacuolation of acinar cells of pancreas and fibrosis was also observed in many cases. Pancreatic atrophy and lesions were found differently in different groups of chicks (Figure 2). In the present study it was noted that stunted chicks of 1–10 days of age (27.78%) and 11–20 days of age (22.22%) might have more lesions in their pancreatic tissues as compared to 21–30-day-old chicks (11.1%) and 31–40-day-old chicks (5.56%) Table 3. Collectively histopathological pancreatic lesions (16.65%) were found in 1–40-day-old stunted chicks, and bleaching of pancreas (47%) in stunted chicks was observed [14]. RSS affected chicks were characterized by white, firm and thin pancreas, exocrine tissues of which may be lost or replaced by fibrous tissues [15]. These findings were in consistent with our observations that exocrine tissues or pancreatic acini was found damaged. Vacuolation was also noted in some of the pancreatic tissues. Section of affected pancreases exhibited the vacuolation of cytoplasm and loss of zymogen granules form acinar cells [17]. Changes were also found in the shape of acinar cells; fibrosis; and pancreatic atrophy in RSS affected birds (Figure 2) [9]. These birds were also showing the signs of retarded feathering and reduced body weights [24]. These findings were in agreement with our results regarding pancreatic lesions. Pancreatic degeneration was also observed in 35% filed cases of more than 13 days of age but in 0.75% of experimental cases of 14 days of age [21]. These observations were not in consisent with our results. Occurrence of lesions may vary depending upon the severity of disease. Low and variable percentage (5–45%) developed pancreatic degeneration in experimental cases of stunted chicks [17]. Pancreatic histopathological changes include the pancreatic degeneration, fibrosis, vacuolation, and loss of zymogen granules from acinar cells in many experimental cases and 54.54% field cases [13]. The structural changes may be found similar, but the greater variations in the chances for occurrence might be due to the fact that experimental cases were given the intestinal homogenates of affected birds so they showed more pancreatic degenerations as compared to our randomly selected field cases that were not provided with any inocula or there may be some nutritional or management variations. Pancreatic degenerations may result in atrophy of pancreases and obstruction of pancreatic ducts. The major source of digestive enzymes is the pancreases and any degenerative change in ducts may disturb the production of normal digestive enzymes resulting in the malfunctioning of digestive processes. Martland [25] described that the pathogenesis of pancreatic lesions seen in stunting syndrome may be due to pancreatic duct obstruction. While studying experimental reproduction of MAS in broilers it was found that pancreatic lesions were exhibited to have less impact on weight gain reduction than intestinal lesions [26].

**Figure 3:**
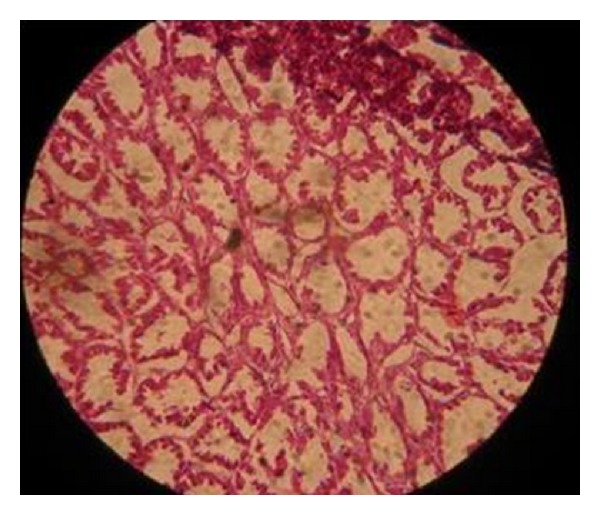
Stunted chick showing bursal lymphocytes depletion.

The histopathological examinations of inflamed proventriculus did not show any specific lesions (Figure 3). In present study the proventricular lesions (5.6%) including dilation of glandular acini were found in 1–40-day-old chicks. 16% proventricular lesions were noted in stunted chicks of G1, while 11% cases with proventricular lesions were found in stunted chicks of G2. Similarly in stunted chicks of G3 5.56% proventricular lesions were observed, while no proventricular lesions were found in stunted chicks of G4. Occurrence of lesions in different age groups may vary depending upon the severity of disease and age of chicks. Enlarged proventriculus in stunted chicks was found in 2% field cases and 5.35% experimental cases. There is no general agreement regarding proventricular lesions in ISS [4, 13, 23, 27], however, many researchers reported that proventriculitis is not consistent in finding histopathological lesions in ISS.

**Figure 4:**
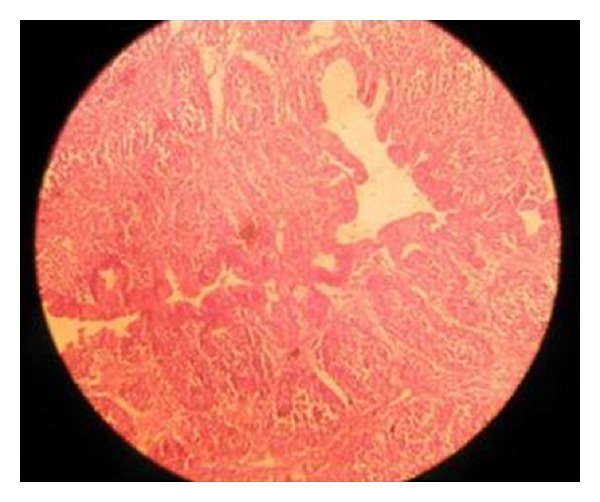
Proventriculus of stunted chick.

Bursa of RSS affected chicks were found atrophied with relatively small follicles and destroyed epithelium. So histopathological examinations of atrophied bursa revealed the degeneration of follicles (43%) in 1–40-day-old chicks. Bursa of stunted chicks was found more atrophied and degenerated in G1 (55.56%) and in G2 (61.1%) whereas stunted chicks of G3 and G4 were found with 38.89% and 16.67% chances for occurrence of lesions. The atrophy of bursa was observed in 42% filed case and 34% experimental cases of RSS while histopathological changes in bursa including cystic dilation of bursal epithelium and depletion of lymphocytes were also seen, (Figure 4) [13]. These results were almost similar to the findings of Montgomery et al. [28].

## 4. Conclusion

RSS is a multifactorial disease. The clinical signs necropsy findings and histopathological lesions in the affected chicken flocks are inter linked. Histopathological lesions may interfere with normal digestive processes and hematological changes may affect the immunity of RSS affected chicks resulting in poor weight gain and delayed or retarded growth. High cull rates, greater than expected variations in weights, reduced weight for age, and sale of small chicks lead to considerable economic losses. Proper vaccination or treatment of affected organs may help reduce the chances of RSS. Appearance and disappearance of RSS is equally sudden making it difficult to decide about the effective control measures. Although there is no satisfactory treatment or vaccination for this disease yet proper poultry house management, strict hygienic and sanitary measures, biosecurity procedures, good parent nutrition, and egg selection reduce the possibility of exposure and incidence of disease. 

## Figures and Tables

**Table 1:** Grouping of experimental chicks.

Sr. no.	No. of groups	Age of chicks (days)	No. of stunted samples in each group *n* = 18	No. of normal samples in each group *n* = 12	Total number of chicks *n* = 30	Total no. of chicks in each group
(1)	G1	4 7 10	6 6 6	4 4 4	10 10 10	30
(2)	G2	11 14 19	6 6 6	4 4 4	10 10 10	30
(3)	G3	22 26 28	6 6 6	4 4 4	10 10 10	30
(4)	G4	32 35 40	6 6 6	4 4 4	10 10 10	30
(5)	G	1–40	72	48	120	120

**Table 2:** % Reduced weights of stunted samples.

Age groups	Average weight of normal chicks *n* = 18 Mean ± SE	Average weight of stunted chicks *n* = 18 Mean ± SE	% reduced weight of stunted chicks
G1	179.67 ± 21.39	66.3 ± 7.11	36%*
G2	503.67 ± 55.07	169.3 ± 11.45	33%*
G3	1164 ± 76.70	329.67 ± 17.14	28.3%*
G4	2058 ± 84.28	658 ± 22.87	31.9%*

*Significant difference.

**Table 3:** Occurrence of histopathological affected organs in each group.

Age groups	Intestinal lesions		Lesions and atrophy of bursa		Lesions and atrophy pancreas		Lesions and atrophy proventriculas	
	Organisms affected with lesions/affected without lesions	% age	Organisms affected with lesions/affected without lesions	% age	Organisms affected with lesions/affected without lesions	% age	Organisms affected with lesions/affected without lesions	% age
G1	8/18	44.44%	10/18	55.56%	5/18	27.78%	3/18	16%
G2	8/18	44.44%	11/18	61.1%	4/18	22.22%	2/18	11%
G3	3/18	16%	7/18	38.89%	2/18	11.1%	1/18	5.56%
G4	2/18	11.1%	3/18	16.67%	1/18	5.56%	0/18	0%
G (1–40 days)	21/72	29%	31/72	43%	12/72	16.65%	6/72	5.6%
